# Magnetic Reversal in Wiegand Wires Evaluated by First-Order Reversal Curves

**DOI:** 10.3390/ma14143868

**Published:** 2021-07-11

**Authors:** Chao Yang, Yuya Kita, Zenglu Song, Yasushi Takemura

**Affiliations:** 1 Department of Electrical and Computer Engineering, Yokohama National University, Yokohama 240-8501, Japan; kita-yuya-ys@ynu.jp (Y.K.); takemura-yasushi-nx@ynu.ac.jp (Y.T.); 2 School of Electrical Engineering, Nanjing Vocational University of Industry Technology, Nanjing 210023, China; zenglu_song@niit.edu.cn

**Keywords:** Wiegand wire, first-order reversal curve (FORC), magnetization reversal, large Barkhausen jump

## Abstract

The magnetic structure of Wiegand wires cannot be evaluated using conventional magnetization hysteresis curves. We analyzed the magnetization reversal of a Wiegand wire by measuring the first-order reversal curves (FORCs). A FeCoV Wiegand wire with a magnetically soft outer layer and a hard magnetic core was used in this study. The magnetization reversal of the soft and hard regions in the wire was identified in the FORC diagrams. The magnetization reversal of the dominantly irreversible process of the soft layer and the magnetic intermediate region between the soft and hard regions was clarified.

## 1. Introduction

A Barkhausen jump, also called the Wiegand effect, refers to the rapid reversal in magnetization of magnetic wires with bistable states [1,2]. Initially, the Wiegand effect was observed in NiFe wires. However, Vicalloy, with a typical composition of Fe_0.4_Co_0.5_V_0.1_, has been identified as the optimum material to realize this effect [3]. Such wires are called Wiegand wires, and they are prepared via twisting and annealing processes [4]. The coercive force on the outer layer of such wires is reduced under the large stress that occurs during the twisting process [5,6], whereas that of the core remains unchanged. Wiegand wires exhibit magnetic structures that can be described as comprising two layers: the magnetically soft outer layer and the hard core, with lower and higher coercive forces, respectively. Fast magnetization reversal in Wiegand wires induces a pulse voltage in a pick-up coil wound around the wire [7]. The amplitude of the voltage can exceed several volts, and the pulse width is approximately 10 μs. When a magnetic field with the appropriate intensity and opposite polarity is applied to Wiegand wires, the magnetization reverses, and a pulse voltage with the opposite polarity is generated.

When an alternating magnetic field is applied to Wiegand wires, positive and negative pulse voltages are repeatedly generated. Thus, Wiegand sensors, consisting of a Wiegand wire and pick-up coil, can be used as self-powered sensors and as power sources for external devices and sensors [8]. Additionally, owing to the fast magnetization of the soft layer, the amplitude of the induced pulse voltage does not depend on the frequency of the applied alternating magnetic field [3,9]. This phenomenon makes Wiegand sensors advantageous over other conventional sensors and power generators. Owing to these specific features, the applications of the Wiegand sensor are no longer limited to generating output signals as a magnetic sensor; instead, it is possible to use it as an electrical power source for various equipment [10]. When used as a power source, Wiegand sensors can charge battery-equipped devices efficiently, thus improving the battery life. Moreover, Wiegand sensors can provide power to devices without internal batteries; this solves the problem of the difficulty in power supply and wiring.

The primary objective of this study was to maximize the application potential of the Wiegand sensor, especially as a power supply to electronics modules. To maximize the power generated by the sensor, it is essential to understand the magnetization process of Wiegand wires. Although the magnetization curves of both the major and minor loops [8] and pulse generation from the wire [11] have been reported previously, the details of the magnetization processes of the two layers in the wire have not been clarified yet. The objective of this study was to clarify the relationship between the magnetization process of Wiegand wires and their magnetic structure. We analyzed the magnetization process using the first-order reversal curve (FORC) method and verified the two-layer magnetic structure without marked boundaries. Herein, we discuss the magnetization reversal of the intermediate layer.

## 2. Materials and Methods

**Figure 1:**
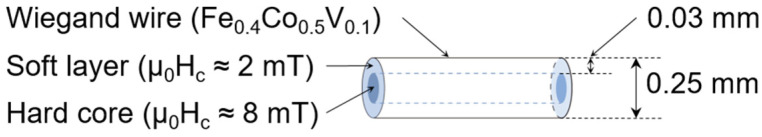
Two-layer magnetic structure of the Wiegand wire.

A Wiegand wire of 5 mm in length and 0.25 mm in diameter, with a composition of Fe_0.4_Co_0.5_V_0.1_ (SWFE, Co. Ltd., Meishan, China), was used in this study. Details of the sample are described elsewhere [8,11]. As the wire was prepared by a twisting process, its outer layer exhibited a lower coercive force than its core. The magnetic structure of the Wiegand wire, with a soft outer layer and hard core, is shown in Figure 1. The coercive forces of the outer layer and core were approximately μ_0_H_c_ = 2 and 8 mT, respectively. Here, μ_0_ denotes the magnetic permeability in a vacuum. This is a conventional structure that was used to interpret the previously reported results [8,11]. As discussed previously [8], and in this study, the coercive force was considered to be gradually changing along the radial direction of the wire. The thickness of the soft layer that exhibited fast magnetization reversal was approximately 0.03 mm [8].

Magnetization curves of normal major and minor hysteresis loops and FORCs were measured using a vibrating sample magnetometer (model 8600 series, Lake Shore Cryotronics, Westerville, OH, USA) at room temperature.

The magnetization data with the applied magnetic field form a class of partial hysteresis curves known as FORCs [12]. FORC analysis is an advanced hysteresis measurement method, which is increasingly being used in research on magnetic materials. This is because it can provide additional information to characterize magnetic properties, which cannot be achieved using normal hysteresis curves [13,14]. FORC information includes the distribution of coercivity and interaction fields and identification of multiple phases in composite or hybrid materials containing more than one phase [13,14,15,16].

**Figure 2:**
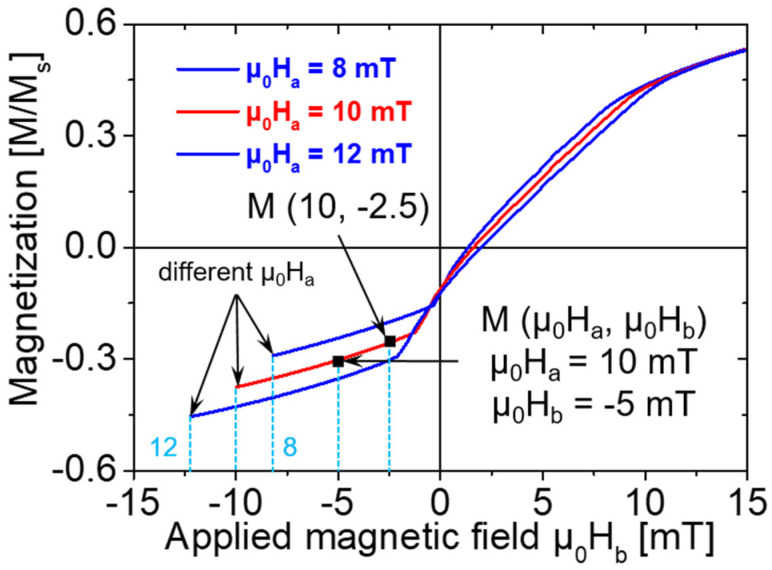
Example of an FORC of a Wiegand wire at different μ_0_H_a_. Two points of magnetization on the FORC, at two applied fields (μ_0_H_b_) with a reversal field (μ_0_H_a_), are represented by M (μ_0_H_a_ = 10 mT, μ_0_H_b_ = −5 mT) and M (μ_0_H_a_ = 10 mT, μ_0_H_b_ = −2.5 mT), respectively.

The FORCs are measured by first saturating the magnetic material in a large positive field (μ_0_H_sat_). Then, the field is decreased to a reversal magnetic field (μ_0_H_a_). The FORC is defined as the magnetization curve measured by increasing the reversal magnetic field (μ_0_H_a_) back to μ_0_H_sat_. Finally, the process is repeated for several values of μ_0_H_a_ to yield a series of FORCs [14]. The magnetization corresponding to any applied magnetic field (μ_0_H_b_) on an FORC can be expressed as M (μ_0_H_a_, μ_0_H_b_), where μ_0_H_b_ ≥ μ_0_H_a_. The parameters μ_0_H_a_, μ_0_H_b_, and M (μ_0_H_a_, μ_0_H_b_) of the FORCs are described in Figure 2.

The FORC distribution is defined as a mixed second derivative:

(1) $$\rho \left(\mu_{0}H_{a},\;\mu_{0}H_{b}\right)=-\frac{\partial^{2}M\left(\mu_{0}H_{a},\;\;\mu_{0}H_{b}\right)}{\partial \mu_{0}H_{a}\;\partial \mu_{0}H_{b}}$$

The calculated FORC distributions are contour plots. To analyze the magnetic properties of the Wiegand wire, {μ_0_H_a_, μ_0_H_b_} and {μ_0_H_c_ = (μ_0_H_b_−μ_0_H_a_)/2, μ_0_H_u_ = (μ_0_H_b_ + μ_0_H_a_)/2} coordinates were used in this work [15,16]. There are several open-source software programs, such as FORCinel [17] and VARIFORC [18], that are available for calculating FORC distributions and plotting FORC diagrams. Advanced functions of the VARIFORC can be selected from the FORCinel menu. Therefore, FORCinel and its auxiliary software (Igor Pro^®^, WaveMetrics Inc., Portland, OR, USA) were used in this study.

## 3. Results

### 3.1. Magnetic Hysteresis Curves

**Figure 3:**
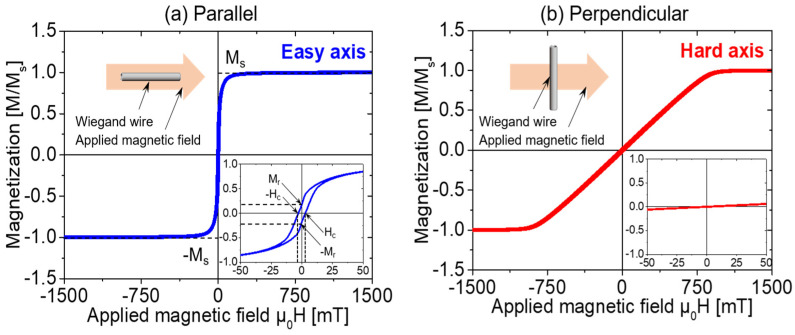
Major hysteresis loops of the Wiegand wire. The applied magnetic field is (**a**) parallel to the wire (easy axis), and (**b**) perpendicular to the wire (hard axis).

Magnetic hysteresis curves have been widely used to characterize the magnetic properties of magnetic materials. To extract comprehensive information on magnetic properties from magnetic hysteresis curves, the major loop was measured with the Wiegand wire parallel and perpendicular to the applied magnetic field in this study, as shown in Figure 3. The normal and magnified views of the major loop in Figure 3a indicate that the sample exhibited remanent magnetization and a coercive field of H_c_ = 3 mT/μ_0_H. These results are in agreement with our previously reported results [8]. When the applied magnetic field was perpendicular to the Wiegand wire, the major loop was significantly narrower, with negligible remanence or coercivity, as shown in Figure 3b. This indicates that the Wiegand wire was anisotropic; the axis along the length was the easy axis of magnetization, and the axis along the diameter was the hard axis of magnetization. Therefore, it was difficult to achieve fast magnetization reversal when the Wiegand wire was perpendicular to the applied magnetic field, and hence, the Wiegand effect was not observed in this case. Consequently, the Wiegand pulse was not induced from the pick-up coil. This observation is consistent with the previously reported results [11].

**Figure 4:**
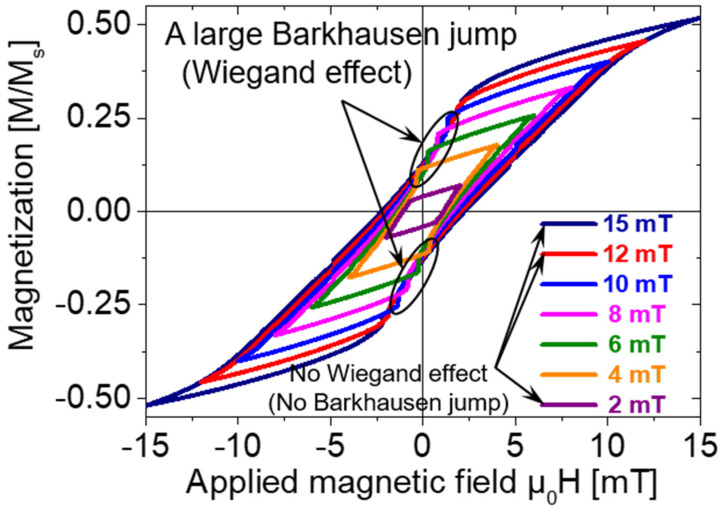
Minor hysteresis loops of the Wiegand wire (easy axis).

Because the Wiegand sensor cannot effectively output energy on the hard axis, we studied only the magnetic properties of the Wiegand wire along the easy axis. The minor loops of the Wiegand wire traced with various maximum amplitudes of the applied magnetic field are shown in Figure 4.

As shown in Figure 4, a fast magnetization reversal was observed in some limited minor loops. When the amplitude of the applied magnetic field was μ_0_H = 2, 12, or 15 mT, no Wiegand effect was observed. The coercive field of the soft layer was approximately μ_0_H = 2 mT [3,8,9]. Therefore, an applied magnetic field equivalent to or less than this value could not reverse the magnetization of the soft layer, and the Wiegand effect was not observed. The coercive field of the hard core was approximately μ_0_H = 8 mT [3,8,9]. It was reported that fast magnetization reversal is not observed under an applied magnetic field larger than the coercive field of the hard core, although the reason for this is not clear. The magnetic field that initiates the fast magnetization reversal of the soft layer is reduced and becomes negative with an increase in the amplitude of the applied field. This is caused by the demagnetizing field in the wire and magnetostatic coupling between the soft layer and hard core, as described later.

### 3.2. First-Order Reversal Curves

**Figure 5:**
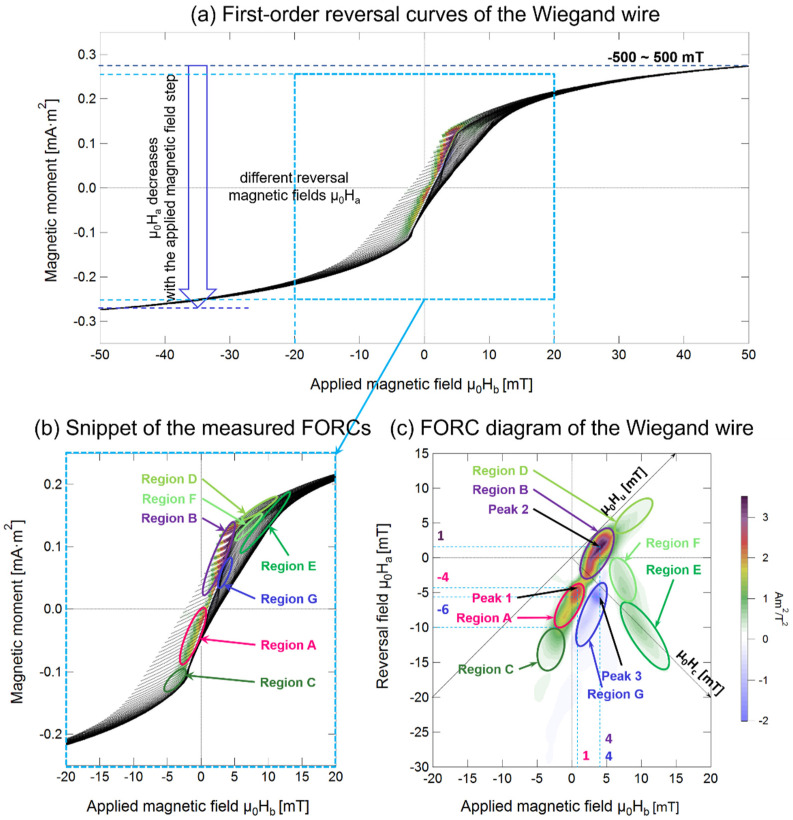
(**a**,**b**) FORCs and (**c**) FORC diagram of the Wiegand wire measured under the applied magnetic field intensity range of −500 to 500 mT. The colors in (**a**,**b**) correspond to the intensity of the FORC distribution in (**c**).

The FORC curves were measured after the magnetization of the sample was saturated to reset its history. In this work, we measured the FORC curves under an applied magnetic field intensity ranging from μ_0_H_a_ = −500 mT to μ_0_H_sat_ = 500 mT. As shown in Figure 3a, the magnetization of the saturated wire was 99.3% (M/M_s_) at μ_0_H_sat_ = 500 mT. Each of the FORC curves was traced by changing the magnetization of the sample by changing μ_0_H_a_ in steps of 0.5. Figure 5a,b shows the part of the measured FORC curves from μ_0_H = −50 to 50 mT and the magnified snippet, respectively. Figure 5c shows the FORC diagram calculated from the FORC curves.

## 4. Discussion

### 4.1. Two-Layer Magnetic Structure and Its Magnetization Reversal

The FORC diagram provides information on the magnetization process and magnetic interaction inside the sample [19,20,21]. As shown in Figure 5c, two spot-like peaks were clearly observed. Peak 1, indicated in the figure, was located at μ_0_H_b_ = 1 mT and μ_0_H_a_ = −4 mT. Because the FORC distribution is calculated using the differential component of the gradient of the magnetization curve using Equation (1), it principally emphasizes an irreversible magnetization of the sample. Peak 1 was broadened to reveal the area indicated as Region A in Figure 5c. This region is also marked in the corresponding FORCs in Figure 5b. Interestingly, it is evident from Figure 5b,c that the reversal fields of the magnetization in Region A were close to the applied alternating magnetic field at which a large Barkhausen jump occurred in the minor loops, as shown in Figure 4. The reversal field corresponding to Region A lies between μ_0_H_a_ = −4 and −10 mT. This field range is consistent with the applied alternating magnetic field of ± 4 to 10 mT, in which the Wiegand effect was observed, as shown in Figure 4. Peak 1 and Region A are attributed to the irreversible magnetization process of the soft axis accompanied by a large Barkhausen jump. Region C showed a continuously broadened distribution related to Region A. The magnetization reversal of the soft layer after the magnetization of the Wiegand wire was saturated in the negative direction by H_b_. As the intensity of the FORC distribution was low, the magnetization process of the soft layer in Region C was almost reversible, and not related to the Wiegand effect.

Peak 2 is located at μ_0_H_b_ = 4 mT and μ_0_H_a_ = 1 mT. Considering that this value of H_b_ lies between those of the coercive fields of the soft layer and hard core, Peak 2 and Region B correspond to the magnetization reversal of the intermediate layer between them or the interaction of the soft layer with the hard core. This magnetization process is irreversible because the intensity of the FORC distribution is high. Region D, with a low intensity FORC distribution, continuously adjacent to Region B, is attributed to the magnetization reversal of the soft layer with an approximately reversible process.

Region E, observed in the range of μ_0_H_b_ > 5 mT and μ_0_H_a_ < −8 mT, is a long and narrow ellipse. It has a wide dispersion along the μ_0_H_c_ axis. This region is attributed to the magnetization reversal of the hard core, as H_b_ and H_a_ were sufficiently larger than the coercive field of the soft layer. The magnetization process is approximately reversible. The origin of Region F, which is centered at μ_0_H_b_ = 8 mT and μ_0_H_a_ = −4 mT, can be attributed to the magnetization reversal of the intermediate layer between the soft layer and hard core, or the magnetization reversal of the partial region of the hard core. This magnetization reversal of the partial region of the hard core is automatically reversed by the corresponding demagnetizing field. Owing to the shape anisotropy, the demagnetizing field in the wire reverses both ends of the wire in the remanent state. The magnetization of the hard core at both ends of the wire is considered to be reversible and does not contribute to the FORC diagram with a negligible intensity.

### 4.2. Interactions between the Two Layers

Although there are different field components of magnetization reversal in the Wiegand wire, when there is no interaction between these components, they generally form a peak or ridge along the zero-bias axis (H_u_ = 0) [22]. However, it can be seen from Figure 5c that these are spread along the H_u_ axis of the FORC diagram of the Wiegand wire. Interactions between components can displace these components off the zero-bias axis and/or can lead to the development of ridges [22]. This indicates that there were interactions between these components, i.e., there were interactions between the soft layer and the hard core of the Wiegand wire.

As shown in Figure 5c, the interaction between the soft layer and hard core displaces the hard core from the zero-bias axis. This interaction can lead to the formation of a ridge, which indicates that Region F belongs to the hard core (Region E). If there is no interaction between the soft layer and hard core, then the FORC distribution that originates from the hard core would be located between Region E and F, instead of spreading out and developing into a ridge. This can also be observed in Figure 5c, wherein the soft layer (Region A) and a part of the hard core (Region F) are not completely separated, and there is an intermediate layer, i.e., Region B, between them. This intermediate layer further indicates that the hard and soft layers interacted with each other. Therefore, it can be concluded that the intermediate layer (Region B) developed because of the interaction between the soft layer and hard core of the Wiegand wire used in this study.

Owing to the presence of the intermediate layer (Region B), it was determined that there was no clear boundary between the two layers in the Wiegand wire. The degree of the magnetization reversal field component spread along the H_u_ axis can be used to measure the strength of the interaction [21,23]. It can be inferred that the interaction between the soft layer and the hard core was strong, as shown in Figure 5c.

### 4.3. Negative Region in the FORC Diagram

**Figure 6:**
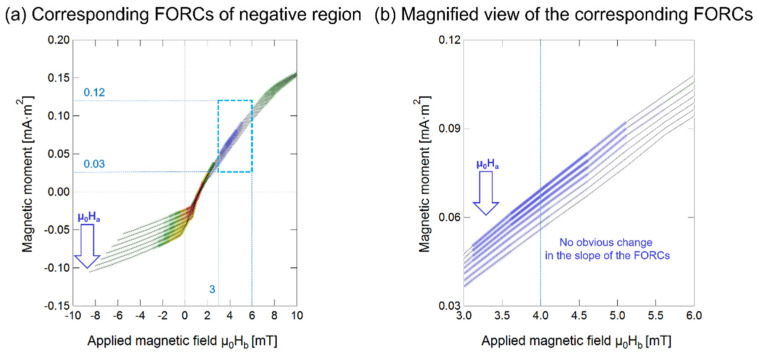
(**a**) FORCs responsible for the negative region (Region C) and (**b**) the magnified view.

It can be seen from Figure 5c that the FORC diagram comprises two distinguishable parts: a positive FORC distribution (ρ (μ_0_H_a_, μ_0_H_b_) > 0) and a negative FORC distribution (ρ (μ_0_H_a_, μ_0_H_b_) < 0). A peak is observed in the negative region (Region G, Peak 3), which is just below Peak 2, and is located at approximately μ_0_H_b_ = 4 mT and μ_0_H_a_ = −6 mT, as shown in Figure 5c. The FORCs responsible for the negative region and its magnified view are shown in Figure 6.

The origin of negative regions commonly seen in FORC diagrams has been discussed previously [24]. It is well-known that the negative region is an intrinsic feature of the FORC diagrams for noninteracting Stoner-Wohlfarth particles [24,25]. The negative region of the FORC diagram of the Wiegand wire cannot be readily explained by this theory as the position of the negative region is located far from the H_u_ axis [24], just below the main peak, as shown in Figure 5c. Carvallo et al. observed a negative region in the FORC diagram of hematite, a magnetite mixture, and a bimodal magnetite mixture sample [26]. However, these diagrams also could not explain the origin of the negative region in the FORC diagram of the Wiegand wire. The Wiegand wire used in this study had a typical composition of Fe_0.4_Co_0.5_V_0.1_, which is not a mixture.

Muxworthy et al. explained the negative regions by the slope change in raw FORCs. Specifically, if the slope increases with decreasing μ_0_H_a_, then the distribution is greater than zero. When there is no change in the slope, the distribution is zero, and if the slope decreases, then the distribution is less than 0 [19,27,28]. Based on this conclusion, the original FORC data of the Wiegand wire were examined. However, the phenomenon of slope change was not observed. At μ_0_H_b_ = 4 mT, each individual FORC curve branch of the Wiegand wire were approximately parallel to each other, as illustrated in Figure 6b. In other words, FORCs parallel to each other indicate that the slope does not change, and the distribution ρ (μ_0_H_a_, μ_0_H_b_) is zero, not less than zero. Therefore, this observation could not explain why the distribution of Wiegand wires in this region is less than zero.

In the FORCs shown in Figure 5a,b, it can be seen that the part with a distribution less than zero appears between the soft layer and the hard core, and there is a strong interaction between the soft layer and the hard core, as discussed previously. Therefore, it can be inferred that the negative region in the FORC diagram of the Wiegand wire also appears due to the interaction between the soft and hard cores. Pike et al. quantitatively showed that these interactions can produce a negative region in the FORC diagram [21,27]. Therefore, it was important to evaluate if the presence of the negative region of the FORC diagram, observed in this work, was due to the interactions between the soft layer and hard core of the Wiegand wire, as explained before.

## 5. Conclusions

In this study, the magnetization properties of a Wiegand wire were examined using FORC diagrams. The magnetic properties of the Wiegand wire were successfully clarified through a detailed analysis of the prominent features in its FORC diagram. It was found that the magnetization process of the soft layer is an irreversible process. The switching field and the intensity range of the applied magnetic field that produces the Wiegand effect with a large Barkhausen jump corresponded well with both the minor hysteresis curves and FORC diagram. The irreversible magnetization process of the soft layer, not accompanied by a large Barkhausen jump, was also observed in the FORC diagram. The magnetization of the hard core was almost reversible. The magnetization of the intermediate region between the soft layer and the hard core, which is also interpreted as the soft layer interacting with the hard core, was observed in the FORC diagram. These results provide evidence for the magnetic structure of the Wiegand wire, which cannot be evaluated using conventional magnetization hysteresis curves. As the Wiegand wire comprises components with different magnetic properties and interactions, FORC analysis is an effective method to further investigate and clarify its magnetic properties.

## Data Availability

Not applicable.
